# CARD8 and NLRP1 Undergo Autoproteolytic Processing through a ZU5-Like Domain

**DOI:** 10.1371/journal.pone.0027396

**Published:** 2011-11-08

**Authors:** Andrea D'Osualdo, Christian X. Weichenberger, Roland N. Wagner, Adam Godzik, John Wooley, John C. Reed

**Affiliations:** 1 Sanford-Burnham Medical Research Institute, La Jolla, California, United States of America; 2 Department of Pharmacology, University of California San Diego, La Jolla, California, United States of America; 3 Center for Biomedicine, European Academy of Bozen/Bolzano (EURAC), Bolzano, Italy; University of Pittsburgh, United States of America

## Abstract

The “Function to Find Domain” (FIIND)-containing proteins CARD8 (Cardinal; Tucan) and NLRP1 (NALP1; NAC) are well known components of inflammasomes, multiprotein complexes responsible for activation of caspase-1, a regulator of inflammation and innate immunity. Although identified many years ago, the role of the FIIND is unknown. Here, we report that CARD8 and NLRP1 undergo autoproteolytic cleavage at a conserved SF/S motif within the FIIND. Using bioinformatics and computational modeling approaches, we detected striking structural similarity between the FIIND and the ZU5-UPA domain present in the autoproteolytic protein PIDD. This allowed us to generate a three-dimensional model and to gain insights in the molecular mechanism of the cleavage. Site-directed mutagenesis experiments revealed that the second serine of the SF/S motif is required for CARD8 and NLRP1 autoproteolysis. Furthermore, we discovered an important function for conserved glutamic acid and histidine residues, located in proximity of the cleavage site in regulating the autoprocessing efficiency. Altogether, these results identify a function for the FIIND and show that CARD8 and NLRP1 are ZU5-UPA domain-containing autoproteolytic proteins, thus suggesting a novel mechanism for regulating innate immune responses.

## Introduction

The NLR (nucleotide-binding domain and leucine-rich repeat containing) gene family is a relatively new class of innate immunity proteins that operate as intracellular surveillance molecules for sensing and responding to exogenous pathogens and endogenous “danger” signals derived from tissue injury[1], [2]. Inactive NLRs are present as monomers, auto-inhibited by their C-terminal leucine-rich repeat (LRR) domains, which control protein oligomerization mediated by their central nucleotide-binding domain, also known as NACHT. Upon oligomerization, signal transduction is propagated by the presence of various effector domains, usually caspase recruitment domains (CARDs) or pyrin domains (PYDs). The presence of different effector domains is also used to further classify NLRs in at least two subgroups: the PYD containing NLRP group and the CARD containing NLRC group. PYD and CARD, together with death domain (DD) and death effector domain (DED), belong to the death fold superfamily domains, comprised of α-helical bundles (typically with 6 anti-parallel α-helices) that form highly specific homotypic interactions among signaling partners[3].

NLRP1 protein differs from all the other NLRP proteins in the C-terminal region, which contains also a FIIND domain followed by a CARD domain, both located after the LRR. The acronym FIIND (domain with function to find) was coined several years ago to indicate a highly conserved protein region with unknown function[4]. Based on amino acid sequence comparisons, the FIIND domain is present in only two proteins encoded in the human genome–NLRP1 and the CARD-containing protein CARD8 (Cardinal; Tucan)[4]. At the genomic level, a nearly identical exon-intron organization of the FIIND-CARD module in both NLRP1 and CARD8 suggests a common ancestral origin of these genes[5]. NLRP1 and CARD8 have been described to form inflammasomes, molecular platforms essential for CARD-CARD-mediated recruitment and activation of caspase-1 and cleavage of pro-IL-1ß[6], [7].Hereditary polymorphisms in both the *NLRP1* and *CARD8* gene of humans have been correlated with autoimmune and chronic inflammatory diseases[8], [9].

In this report, we demonstrate that the FIIND is a previously unrecognized type of ZU5-UPA domain, which undergoes post-translational autocleavage. The findings extend intra-molecular autoproteolysis mediated by the ZU5-UPA motif to the NLR family of innate immunity proteins.

## Results and Discussion

### CARD8 and NLRP1 undergo cleavage within the FIIND

**Figure 1:**
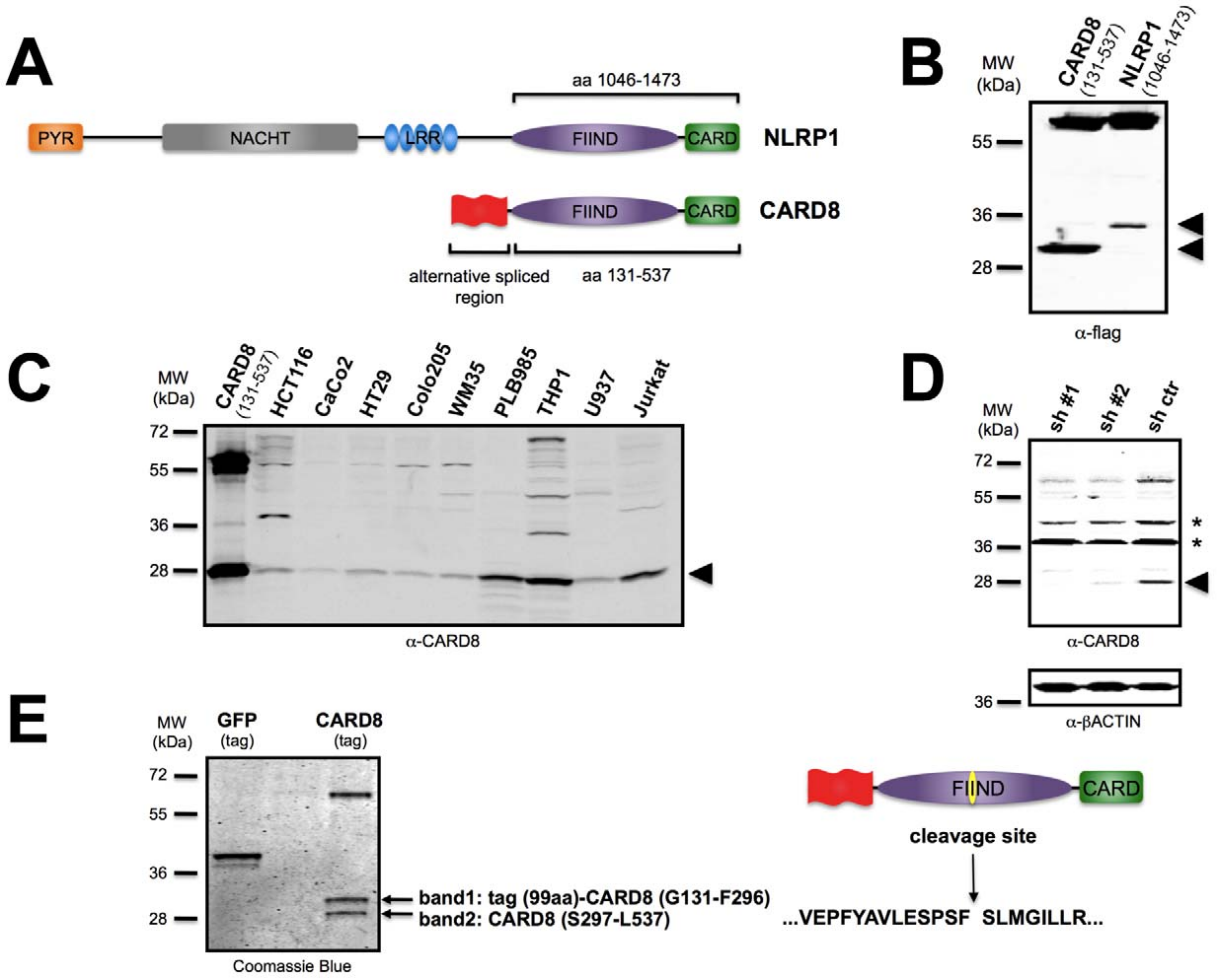
CARD8 and NLRP1 undergo cleavage within the FIIND. (A) Schematic representation of NLRP1 and CARD8 domains structure. The CARD8 region for which numerous isoforms arise through alternative mRNA splicing is indicated in red. The regions of the CARD8 and NLRP1 proteins that contain the FIIND and CARD and that were expressed in cells are shown. (B) HEK293T cells were transiently transfected with plasmids encoding the FIIND-CARD region of CARD8 (*left*) or NLRP1 (*right*) with N-terminal flag tags. After 24 hours, cell lysates were prepared and analyzed by SDS-PAGE/immunoblotting using anti-flag antibody. Unexpected smaller forms of the flag-tagged proteins are indicated by arrow heads. Molecular weight markers are indicated in kilo-Daltons (kDa). (C) Analysis of endogenous CARD8. Cell lysates from the indicated cancer and leukemia cell lines were normalized for total protein content and subjected to SDS-PAGE/immunoblot analysis using rabbit antiserum directed against the C-terminal portion of CARD8. The smaller form of CARD8 is indicated by arrowhead. (D) HCT116 cells were stably transduced with lentiviruses encoding either two different shRNAs or scrambled control. Cell lysates were analyzed by immunoblotting using a rabbit anti-CARD8 antibody. Full-length CARD8 migrates at molecular mass of ∼60 kD, whereas cleaved CARD8 appears at ∼28 kD (arrowhead). As a loading control, membranes were re-probed with anti-ß-actin antibody. Asterisks indicate non-specific bands. (E) HEK293T cells stably expressing SBP-tagged proteins were lysed and incubated with magnetic streptavidin beads, followed by elution with biotin. Eluted proteins were subjected to SDS-PAGE and stained with Comassie Blue. The bands indicated by an arrow (band 1; band 2) were subjected to tryptic digestion and analyzed by LC-MS to reveal peptides corresponding to a novel cleavage site between Phe-296 and Ser-297.

During attempts to identify protein interaction partners of the FIINDs of CARD8 and NLRP1, we noticed the appearance of unexpected protein products when expressing constructs in HEK293T cells encoding the FIIND-CARD regions of CARD8 and NLRP1 (Figure 1A, B). Specifically, for CARD8, we observed the appearance of two bands as determined by SDS-PAGE analysis–one at the predicted size of ∼60 kDa and another of ∼30 kDa (Figure 1B). The smaller band was also observed upon transfection in other cell lines and in the presence of broad-spectrum caspase inhibitor z-VAD (data not shown). Similarly, expression in HEK293T cells of the homologous FIIND-CARD region of NLRP1 (1046-1473) also resulted in two protein products, one migrating at the expected size of ∼60 kDa and another at ∼34 kD (Figure 1B).

Considering that the smaller forms of these proteins could represent an artifact, we next checked the status of the endogenous CARD8 protein by performing immunoblot analysis of cell lysates. Thus, using an antiserum raised against the last 50 amino acids of CARD8, we tested several tumor cell lines of epithelial and hematopoietic cell lineages. In all cases, a prominent ∼28 kDa form of the CARD8 was present, typically (but not always) in addition to the expected full-length ∼60 kDa CARD8 protein, as well as other bands detected in some cell lines (Figure 1C).

To verify the identity of the smaller form of CARD8, shRNA-mediated gene silencing was used. For these experiments, human HCT116 cells were infected with two different shRNAs targeting CARD8 mRNAs or with a scrambled control, then lysates were prepared and CARD8 protein levels were analyzed by immunoblotting. As shown in Figure 1D, not only did the ∼60 kDa band corresponding to full-length CARD8 show reduced levels in cells infected with the CARD8-targeting shRNAs compared to the scrambled control, but the levels of the ∼28 kDa form of CARD8 were also reduced. Again, this antibody is directed against the C-terminal region of CARD8 and thus would not detect the N-terminal cleavage product. Collectively, these results suggest that the smaller form of CARD8 does not represent an artifact of over-expression of CARD8.

We utilized mass spectrometry to analyze the smaller form of CARD8. To this end, we generated stable HEK293T cells expressing either CARD8 or GFP (negative control) with a N-terminal streptavidin binding peptide (SBP) tag. These proteins were purified by a streptavidin pull down followed by biotin elution, and analyzed by SDS-PAGE with Coomassie Blue staining (Figure 1E). In the case of CARD8, three protein products were detected, including the ∼60 kDa full-length protein and two smaller fragments of ∼30 and ∼28 kDa. The simplest interpretation of these results is that CARD8 undergoes proteolytic cleavage to produce two smaller fragments.

The two smaller bands were subjected to tryptic digestion and analyzed by mass spectrometry. As expected, both bands corresponded to fragments of CARD8. Two of the recovered peptides–VEPFYAVLESPSF and SLMGILLR–were of interest, as they could not have been generated by trypsin cleavage (Figure S1), thus identifying Phe-296/Ser-297 as the cleavage site within CARD8 (Figure 1E). Importantly, although only the N-terminal CARD8 fragment contained the SBP tag, both fragments were pulled-down by streptavidin, indicating that they remain associated after proteolytic processing (Figure 1E).

### The FIINDs of CARD8 and NLRP1 are predicted to be ZU5-UPA domains, with similarity to the UNC5b, PIDD and Ankirins proteins

Since the cleavage site was identified in the middle of FIIND, we searched for structural templates by applying bioinformatics tools. Using the entire CARD8 protein sequence for finding distantly related protein structures, HHpred software gave a significant hit with UNC5b[15]. Indeed, a high confidence prediction can be made for the region corresponding to the cytoplasmic portion of UNC5b, namely a ZU5 domain (as initially found in ZO-1 and UNC5), followed by a UPA domain (conserved in UNC5, PIDD and Ankirins) and by a Death Domain (DD) fold[15]. Interestingly, the same domain organization is also present at the C-terminus of PIDD, a LRR containing protein controlling apoptosis and NF-κB activation[25], [26]. From our structural model it follows that both NLRP1 and PIDD adopt the same domain configuration, with the ZU5 domain included between a LRR and a DD superfamily fold. Remarkably, PIDD undergoes autocleavage processing immediately after each of its ZU5 domains at a highly conserved HF/S sequence, similar to the SF/S sequence we found in CARD8 and NLRP1[27].

Based on the reported alignment, a three-dimensional model of the protein was constructed with the Modeller tool and refined with the Relax program of the Rosetta suite, which improved the packing of side-chains and arrangement of secondary structure motifs. The resulting model was used to study both the cleavage site and the interface between the ZU5 and CARD domains. The region of CARD8 containing the FIIND and CARD can be divided into three domains. Following the nomenclature of the UNC5b template, these three domains consist of (1) the all-beta strand ZU5-like domain (residues Leu-162 to Thr-309), (2) the mainly beta strand UPA-like domain (residues Arg-310 to Pro-434), followed by a linker region of about 20 amino acids, and finally (3) the all-alpha helix CARD domain (residues Lys-455 to the end). In our model, the FIIND encompasses both the ZU5-like and UPA-like domains. The N-terminal ZU5-like domain is located between the UPA-like and the CARD domains. Similar results were obtained with the FIIND-CARD region of NLRP1 (refseq accession code NP_127497.1), with the exception that the UPA-like domain was aligned with less confidence by the HHpred algorithm. However, the location of the cleavage site within the ZU5-like domain can be predicted with high confidence.

**Figure 2:**
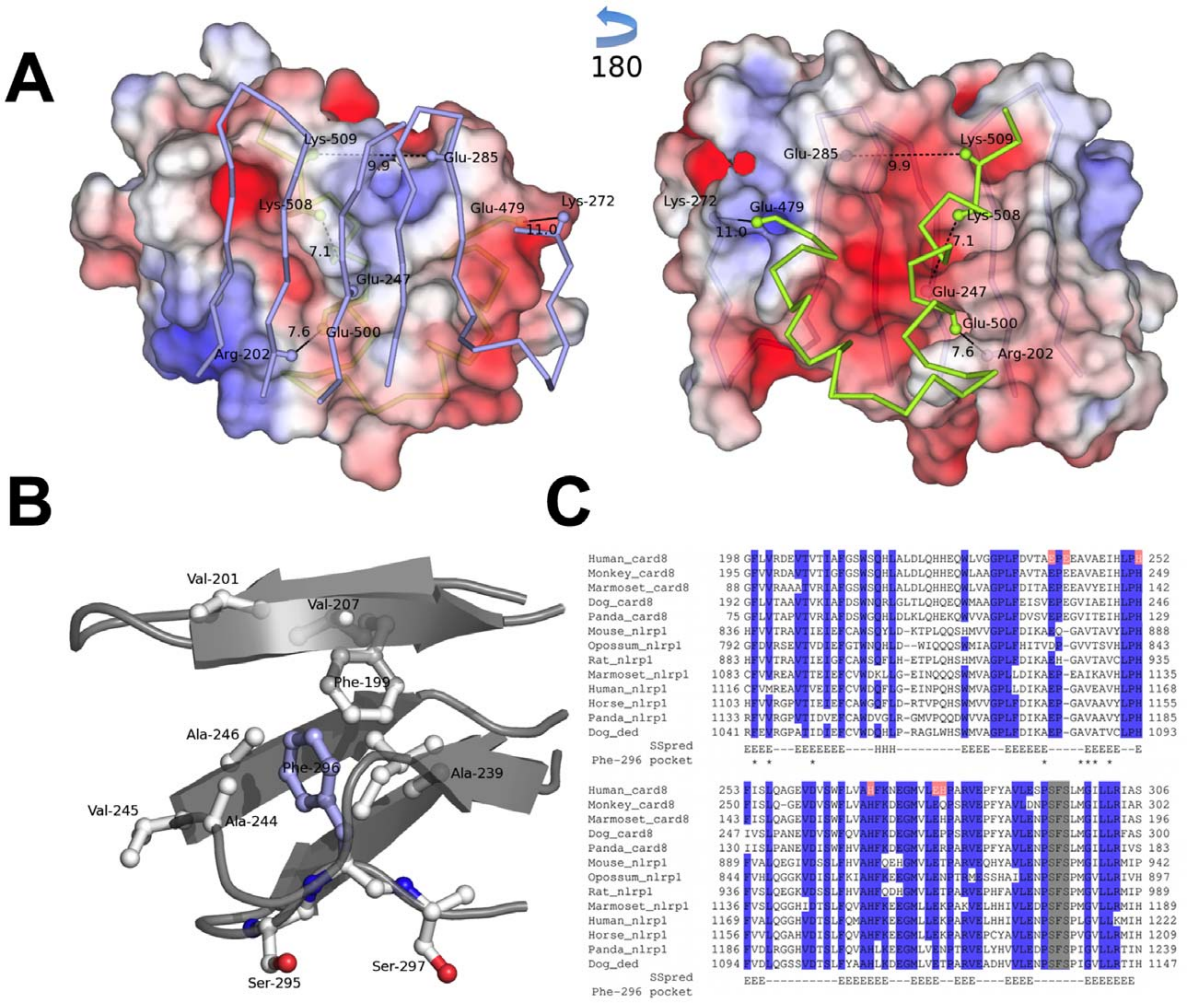
CARD8 three-dimensional model. (A) Charge distribution and predicted salt bridges in the interface between the ZU5-like and the CARD domain. Only interface forming segments of the protein chain are shown as a C-alpha atom connecting trace. Atoms of the ZU5-like domain are colored light blue, those belonging to the CARD domain are colored green. Putative salt bridges are indicated by a dashed line connecting the C-beta atoms of the involved residues, labeled with their distance in units of Ångströms. The left side of the figure presents the electrostatic surface potential of the CARD domain facing the ZU5-like domain, whereas the right side is obtained by a 180-degree rotation and displays the surface of the ZU5-like domain. Surfaces are rendered semitransparent such that the backbone of the opposite domain is visible. The charge distribution ranges from -5 (red) to +5 (blue) in units of *K_b_T/e_c_*, where *K_b_* is Boltzmann's constant; *T = 310*, temperature; and *e_c_*, charge of an electron. Compared to the ZU5/death domain interface of the template structure, this model lacks a clear hydrophobic patch and exhibits a higher degree of electrostatic interactions. (B) Hydrophobic pocket for the phenyl ring of Phe-296. Burial of the phenyl ring is an important structural feature of the SFS motif as it positions its neighboring serine side-chains for activation. Seven of the eight hydrophobic residues shown in this figure are thoroughly conserved in all FIIND domains. The exception is Ala-246, which, according to the multiple sequence alignment (see below), can be substituted by a variety of amino acids. However, each of them is principally able to contribute hydrophobic interactions with their carbon side-chain atoms. The C-alpha trace of the protein chain is rendered in dark grey cartoon representation. Atoms are colored by their element, carbon, white; nitrogen, blue; and oxygen, red. The buried phenylalanine residue is highlighted in light blue and important side-chains are rendered as a ball-and-stick model. (C) Multiple sequence alignment (MSA) for selected protein sequences containing the FIIND domain. Proteins with sequence identities higher than 90% are not shown. As a query human CARD8 FIIND domain (Human_card8) was taken to detect homologs in the RefSeq database using NCBI Blast. The resulting sequences were trimmed to highlight important residues from the FIIND domain. (The complete MSA is provided in Methods S1.) Protein sequences are sorted by sequence identity to Human_Card8, giving two distinct clusters of FIIND domains, one for CARD8 proteins and the other for NLRP1 proteins, which is also reflected when phylogenetic trees are constructed from these sequences (data not shown). A grey box highlights the SFS motif. See Methods S1 for additional details. Human_card8 residues selected for mutagenesis experiments are colored orange. Amino acids printed on blue background signify columns with residue identities greater than or equal to 90%. Secondary structure prediction for Human_card8 was performed with Psipred shown in the row labeled “SSpred” containing the following symbols: dash (−), predicted coil; E, predicated strand; and H, predicted helix. In a second row termed “Phe-296 pocket” residues are marked with an asterisk (*), which form the hydrophobic pocket for the phenyl ring of Phe-296. Looking at these columns, this pocket is highly conserved across all FIIND domains, except for Ala-246, which is replaced by various non-hydrophobic amino acids. Protein sequence names provide common names for the organism and the type of protein. Organisms are abbreviated as follows: Human, *Homo sapiens*; Monkey, *Macaca mulatta*; Marmoset, *Callithrix jacchus*; Panda, *Ailuropoda melanoleuca*; Mouse, *Mus musculus*; Opossum, *Monodelphis domestica*; Rat, *Rattus norvegicus*; Horse, *Equus caballus*; Dog, *Canis familiaris*; Suffices can be card8, CARD8-like proteins; nlrp1, NLRP1-like proteins; and ded, death effector domain containing proteins.

The distinct interface between the ZU5 and the DD in the UNC5b protein structure appears to be partially retained in our model of the CARD8 protein. Figure 2A provides insight into the interface from two views, observing the ZU5-like domain from the CARD domain and vice versa. Similar to the template structure, the interface buries approximately 800Å^2^, but is predicted to be less hydrophobic in CARD8. In this regard, the FIIND/CARD interface in CARD8 shows stronger electrostatic potential, including four possible salt bridges between the two domains: (1) Glu-247↔Lys-508, (2) Arg-202↔Glu-500, (3) Lys-272↔Glu-479, and (4) Glu-285↔Lys-509, where only the last of these is conserved in UNC5b (involving residues Glu-666↔Arg-913). One example of hydrophobicity change is given by amino acid Val-619 in UNC5b, which in our CARD8 model is replaced by Glu-247, an amino acid predicted to participate in a salt bridge in CARD8. Finally, we observed a considerable change in the cysteine composition between the CARD8 model and the UNC5b template. The UNC5b protein contains a total of 13 cysteine residues and one disulphide bond formed by Cys-663↔Cys-670. In contrast, the FIIND and CARD domains completely lack cysteine residues. The disulphide bond-forming cysteines have been replaced by two alanine residues and create a small hydrophobic patch. Cysteine residues are only found at the N-terminus of the CARD8 protein prior to the FIIND in a region, which, according to the Xtalpred server[28], is characterized by low complexity and high disorder.

The cleavage we observed experimentally occurs between residue Phe-296 and Ser-297 in the completely conserved SFS motif of the FIIND. These residues are located at the C-terminus of the ZU5-like domain just proximal to a long beta strand that has been shown to be crucial for fold stability of UNC5b[15]. The distinguishing feature of this SFS motif is the deeply buried phenylalanine residue Phe-296, highlighted in Figure 2B, which has previously been described in the autocleavage HFS motif of Nup98[29]. The motif is located on a loop and the phenyl ring points into a pocket created by the side-chains of several hydrophobic residues distributed across two layers of a beta sandwich. This hydrophobic core is completely conserved between the UNC5b template and the CARD8 model. The essential Phe-296 residue corresponds to Leu-677 in UNC5b. All hydrophobic residues shown in Figure 2B have a hydrophobic counterpart in the template structure.

### Confirmation of the autocleavage sites of CARD8 and NLRP1 by mutagenesis

In contrast to enzymatic proteolysis, which is defined as an inter-molecular reaction involving an enzyme, autoproteolysis is characterized by a peptide bond disruption caused by an intra-molecular reaction[30]. The proposed mechanism for PIDD self-cleavage is similar to other autoproteolytic proteins such as Nup98, Inteins, Pyruvoyl enzymes, N-terminal nucleophile (Ntn) hydrolases, and Hedgehog proteins[27], [29], [30]. These autoproteolytic processing events are initiated by a nucleophilic attack of a serine, threonine, or cysteine residue at the preceding scissile peptide bond, leading to an N-O or N-S acyl rearrangement of the peptide bond (acyl shift) and resulting in a more reactive (thio)ester bond[29], [31]. Attack of the ester intermediate by a second nucleophile (usually water) results in cleavage (hydrolysis) of the peptide bond between serine, threonine, or cysteine and the preceding amino acid. In many autocleaved proteins, the cleaved fragments remain associated after processing[27]. This seems also to be the case for CARD8, where the hypothetical interaction between the ZU5-like domain and the CARD (see model; Figure 2) could explain why the CARD8-C fragment was pulled-down with the tagged CARD8-N fragment (Figure 1D).

**Figure 3:**
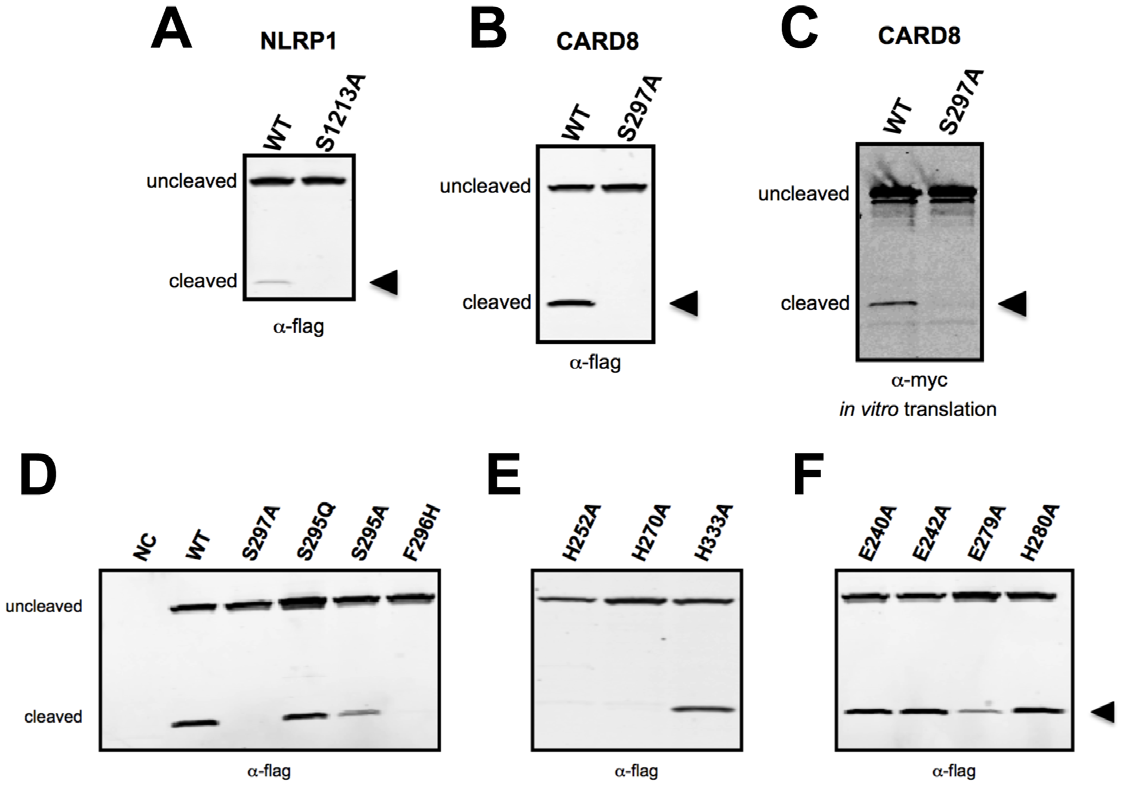
Mutagenesis analysis of CARD8 and NLRP1 autoproteolytic mechanism. In (A), (B), and (D), HEK293T cells were seeded in 6-well plates at a density of 1×10^6^ cells per well and transiently transfected with 2 µg of indicated expression plasmids. After 24 hours, cells were collected and lysed. Cleared lysates were normalized for total protein content and subjected to SDS-PAGE/immunoblot anlaysis using anti-flag antibody. Plasmids transfected were (A) flag-NLRP1 and flag-NLRP1(S1213A), (B) flag-CARD8 and flag-CARD8(S297A), and (D) various flag-tagged CARD8 mutants. WT  =  wild-type CARD8; NC  =  non-transfected control. (C) *In vitro* translation of myc-tagged CARD8 and CARD8(S297A) was performed and equal-volume aliquots of the reaction were subjected to SDS-PAGE/immunoblotting using anti-myc antibody. Arrowheads indicate cleaved protein fragments.

The similarities of the CARD8 (SF/S) and PIDD (HF/S) cleavage sequences, together with our three-dimensional model of the FIIND/ZU5-like domain, prompted us to investigate whether CARD8 is subject to an autoproteolytic mechanism analogous to PIDD. Additionally, we used mutational analysis to clarify whether the analogous Ser-1213 in the SF/S sequence of NLRP1 is required for its autocleavage. Substitution of Ser-1213 of NLRP1 or Ser-297 of CARD8 with alanine completely abrogated NLRP1 and CARD8 cleavage in HEK293T cells (Figures 3A, B). Thus, these results suggest that Ser-1213 of NLRP1 and Ser-297 of CARD8 are functionally equivalent to Ser-446 and Ser-588 of PIDD, which are essential for the nucleophilic attack during autocleavage. Similar results were obtained by comparing *in vitro* translated wild-type CARD8 and S297A CARD8 (Figure 3C), further indicating that a cellular protease is unlikely to be responsible for the observed cleavage of CARD8.

The importance of the conserved SFS motif in CARD8 was further investigated by mutagenesis of the Phe-296 residue. In both Nup98 and PIDD, the corresponding phenylalanine was described to play an important structural role and its substitution with histidine dramatically abolished autocleavage[27]. For this reason we used the same mutagenesis strategy, substituting the Phe-296 for histidine. Indeed, the F296H mutant of CARD8 was severely defective in autocleavage activity (Figure 3D). These results, together with the predictions of our three-dimensional model (Figure 2) suggest that, similar to the corresponding residue in PIDD, Phe-296 of CARD8 is important for providing a structural anchor to maintain the distorted backbone conformation. Interestingly, the predicted structure of the CARD8 FIIND implies that the presence of a hydrophilic histidine instead of phenylalanine could not be buried inside the hydrophobic pocket between the two layers of a beta sandwich (Figure 2B), and thus histidine (unlike phenylalanine) would not produce the necessary strain on the polypeptide backbone required for autocleavage.

### Conserved histidine residues play an important role in CARD8 cleavage

Having verified the importance of the Phe-296 and Ser-297 residues in the SF/S motif of CARD8, we turned to the role of Ser-295, which substitutes for the functionally important histidine residue present in the HFS sequence of PIDD and Nup98. Both mutagenesis experiments and the three-dimensional structure showed that Nup98 mutations H862Q or H862A only slightly affected protein autocleavage[32]. Consequently, we evaluated the role of CARD8 Ser-295, by performing the same mutagenesis experiment. S295A showed only a small reduction in autocleavage (in line with the results obtained in Nup98 for the H862A mutant), while S295Q had no impact on CARD8 autocleavage (Figure 3D). Together these results suggest that, although marginally involved in CARD8 autoprocessing, Ser-295 is not the only contributor to Ser-297 activation during the cleavage reaction.

To find functionally relevant amino acids for the deprotonation and activation of the nucleophilicity of Ser-297, we searched the ZU5-like domain for amino acids with high conservation across different species (Figure 2C and Figure S2). Only three histidines, His-252, His-270 and His-333 were conserved in the multiple sequence alignment. Although none of them was located in proximity of Ser-297 according to our model (Figure S3), mutagenesis experiments revealed that both His-252 and His-270, but not His-333, are crucial for CARD8 autoprocessing (Figure 3E). The side chain of His-252 forms a hydrogen bond to the backbone carbonyl oxygen of Glu-285, forcing its side chain into a direction that promotes formation of a stabilizing salt bridge with a residue from the CARD domain. Thus, dependence on His-252 for autoproteolytic activity is probably related to protein stability considerations, and unlikely to reflect a direct role for His-252 in proteolysis. Interestingly, despite the low sequence similarity between our CARD8 model and the UNC5b template structure, both His-252 and Glu-285 are conserved as residues His-624 and Glu-666 in UNC5b protein, where the latter residue is also involved in the formation of a salt bridge to the death domain (See Figure S4). Residue His-270 is located in a less reliable part of the model. However, it follows from the template structure that this region forms a complementary beta-sheet of a sandwich. In this case, it is likely that His-270 becomes located near the scissile bond and therefore participates in autocleavage.

Finally, we explored other conserved amino acids that could contribute to Ser-297 nucleophilic activation. Since in various Ntn hydrolases, such as glycosylasparaginase (GA) and Taspase1, an aspartic acid was demonstrated to be essential for autocleavage[33], [34], we focused on possible acidic amino acids around the SFS region. Three glutamic acids were predicted to be spatially close to the SFS region. Specifically, Glu-240 and Glu-279 were selected for mutagenesis experiments because of their conservation. In addition, because it is not conserved in the NLRP1 protein, Glu-242 was selected as negative control, as well as a non-conserved histidine residue next to Glu-279 (His-280). As shown in Figure 3F, among all these mutants, E279A was the only alanine-substituted residue that significantly reduced protein autocleavage. Based on this observation, we propose that Glu-279 modulates CARD8 autoproteolytic activity.

### Conclusions

In summary, our results show that CARD8 and NLRP1 join the repertoire of proteins capable of intra-molecular autoprocessing. We show that Ser-297 in CARD8 and the equivalent Ser-1213 in NLRP1 are responsible for the nucleophilic attack at the scissile peptide bond and therefore essential for protein autoprocessing. Our data also strongly suggest that His-270 in CARD8 is a likely candidate for Ser-297 activation. Furthermore, Ser-295 and Glu-279, together with His-252 and Phe-296, are also important for CARD8 autocleavage, presumably by providing the required structure-related conformational strain necessary for hydrolysis of the peptide bond between Phe-296 and Ser-297 (Figure S5). Elucidating the functional role of the autoproteolytic cleavage will require further investigation, however, we predict this unusual post-translational cleavage plays an important role in regulating inflammasome activity and thus innate immune responses. Finally, we propose that the term FIIND be abandoned, and henceforth, that the corresponding regions of CARD8 and NLRP1 be recognized as ZU5- and UPA-like domains.

## Materials and Methods

### Reagents, Plasmids, and Antibodies

Biotin (B4501) and shCARD8 lentiviral vectors (TRCN118329 and TRCN118331) were purchased from Sigma. For protein purification and LC/MS analysis, the FIIND-CARD region of CARD8 (NP_001171829.1, residues 131-537) was cloned into a N-terminal streptavidin binding peptide (SBP) lentiviral vector using a Gateway^®^ cloning technology. For all other expression studies, FIIND and CARD domains of CARD8 (residues 131-537) and NLRP1 (NP_127497.1, residues 1046-1473) were cloned into a pcDNA3 vector modified to contain a N-terminal flag-tag, followed by a Tobacco Etch Virus (TEV) protease cleavage sequence, with a myc-tag and a flexible linker region of poly-Glycine. All constructs were verified by DNA sequencing. CARD8 antibody was purchased from Abcam (ab24186).

### Cell lines and culture

HEK293T, HCT116, CaCo2, HT29, Colo205, WM-35, THP-1, U937 and Jurkat cell lines were purchased from the American Type Culture Collection (ATCC). PLB-985 cell line was kindly provided by Dr. Mary Dinauer (Washington University School of Medicine, St. Louis, MO).

HEK293T, HCT116, CaCo2, HT29, Colo205 and WM-35 cells were cultured in DMEM media supplemented with 10% fetal bovine serum, 100 U/mL penicillin, and 100 mg/mL streptomycin. PLB-985, THP-1, U937 and Jurkat cells were cultured in RPMI 1640 media supplemented with 10% fetal bovine serum, 100 U/mL penicillin, and 100 mg/mL streptomycin. Media and supplements were purchased from Invitrogen.

### Computational Modelling

The protein sequence of human CARD8 (refseq id NP_001171829.1, isoform a) was selected as a query for a NCBI Blast online search. In this isoform of CARD8, the FIIND is comprised of residues from Leu-162 to Ala-444, followed by the CARD domain, which terminates the protein sequence at residue Leu-537. The resulting list of homologs included sequences for CARD8-like proteins and NLRP1 proteins from several species, including *Pan troglodytes, Mus musculus, Canis familiaris,* and *Ailuropoda melanoleuca*. For each organism, a single gene product was chosen from this hit list, giving priority to isoforms “1” or “a”, where available. Using the resulting 23 protein sequences, a multiple sequence alignment (MSA) was constructed with the Mafft v6.821b[10] program. Belvu alignment viewer software[11] was used for editing and removing sequences with more than 90% sequence identity to another sequence in the MSA. Visualization, coloring, and annotation of the MSA were carried out with Jalview v2.6.1[12].

The complete CARD8 protein sequence was submitted to the HHpred server[13], [14] for remote homology detection. Among many hits to CARD and death domains from the C-terminus of the CARD8 protein, we also found a significant hit to the netrin receptor UNC5b (PDB accession code 3g5b[15]), a protein structure with three distinct domains. The sequence identity for this alignment is only 12% (*E*-value 0.028) but the PsiPred secondary structure prediction[16], [17] was encouraging, in perfect agreement with the DSSP[18] assignment of the template structure.

Based on the CARD8/UNC5b alignment calculated by HHpred, we derived a three-dimensional model with Modeller v9.9[19], [20]. Investigation of this first model indicated a slightly misaligned region at the C-terminus of the CARD domain, resulting in a broken alpha helix. Therefore, the short fragment spanning residues Glu-507, Lys-508, and Lys-509 was corrected manually. Further post-refinement of this initial model was carried out with the Relax program of Rosetta[21], [22] suite v3.2 (codename minirosetta) using standard parameters and an activated wobble option that allowed the backbone and side-chains to adopt a more realistic packing. Figures were generated with PyMOL and electrostatic surface potential computation was carried out with APBS software[23].

### Protein purification

Approximately 20 million of stably-transfected HEK293T cells were suspended in 2 ml of lysis buffer (10% (v/v) glycerol, 50 mM HEPES-NaOH pH 8.0, 150 mM NaCl, 2 mM EDTA, 0.1% (v/v) Igepal CA-650, 2 mM DTT, complete mini protease inhibitors cocktail, 10 mM NaF, and phosphatase inhibitors cocktail) and incubated overnight with magnetic streptavidin beads (Invitrogen). Biotin was used for elution at a final concentration of 10 mM.

### Cell Transfections and Immunoblot analysis

HEK293T cells were seeded into 6-well plates at a density of 1×10^6^ cells per well and transiently transfected with 2 µg of various expression plasmids. After 24 hours, cells were collected and lysed in 10% (v/v) glycerol, 50 mM HEPES-NaOH, pH 8.0, 150 mM NaCl, 2 mM EDTA, 0.1% (v/v) Igepal CA-650, 2 mM DTT, complete mini protease inhibitors cocktail, 10 mM NaF, and phosphatase inhibitors cocktail. Cleared lysates were normalized for total protein content and aliquots were subjected to SDS-PAGE/immunoblot analysis. Epitope-tagged proteins were revealed using anti-flag antibody (Sigma) and using Licor Odyssey system (LiCor).

### Lentiviruses and cell tranductions

The shRNA lentivirus plasmids were packaged by transfection into 293T cells, as described in Krieg et al[24]. HCT116 cells were stably transduced with two different shRNAs targeting CARD8 mRNA or a control shRNA (scrambled RNA). Stably transduced cells were selected in McCoy's medium containing puromycin (5 µg/ml).

### Site-directed Mutagenesis and in vitro translation (IVT)

All mutants were generated by site-directed mutagenesis using QuikChange^®^ II XL kit from Agilent Technologies (Cat# 200521). All primers were obtained using the QuickChange^®^ primer design program. All plasmid constructions were verified by DNA sequencing. In vitro translation of wild-type (wt) and S297A CARD8 constructs were obtained using a TNT^®^ T7 quick coupled transcription and translation system from Promega (Cat# L1170).

## Supporting Information

Figure S1 ***MS2 spectra of band 1 and 2.*** MS2 spectra for peptide VEPFYAVLESPSF from Gel Band 1 with MH+ 1484.7313, m/z 742.8693 and MS2 spectra for peptide SLMGILLR from Gel Band 2 with MH+ 918.5447, m/z 459.7760. b ions (blue) and y ions (red) are corresponding to tandem CID fragmentation of peptides matching the theoretical fragmentation pattern. Yellow M shows the oxidation of methionine.
(TIF)

Figure S2 ***Complete multiple sequence alignment of FIIND domains.*** High residue conservation is found across the whole domain. Sequences are sorted by percentage identity to the first listed protein, Human_card8. The most remote homolog is given by the death effector domain of dog, Dog_ded, which shares 47% identical residues with Human_card8. In a certain sense the opossum FIIND domain presents an outlier since it is equipped with an insertion of 41 residues at the C-terminus. As this fragment is absent in Human_card8, secondary structure prediction is not shown in the ‘SSPred’ row.
(TIF)

Figure S3 ***Complete model of the FIIND and CARD domains of human CARD8 protein.*** The model is based on the structure of UNC5b, deposited in PDB with accession code 3g5b. The FIIND domain consists of two domains, at the N-terminus a ZU5-like domain (green) followed by a UPA domain (blue). The CARD8 protein is terminated by a CARD domain (magenta). The center of the figure displays in ball-and-stick mode the SFS motif given by residues Ser-295, Phe-296, and Ser-297. Furthermore, residues Glu-240, Glu-242, His-252, His-270, Glu-279, His-280, and His-333 were selected for mutagenesis studies, they are colored orange in the MSA (Figure 2C and Supplementary Figure 2) and are also visualized as ball-and-stick representations. Autocleavage takes place at the C-terminus of the ZU5-like domain right before the last beta strand, which has been shown to be crucial in UNC5b. (In this figure, this beta strand has been rendered as an arrow for reasons of clarity even though the model lacks a beta sheet containing the strand.) A certain portion of the model lacks a confident structural prediction and has been rendered black. We assume this part forms another beta-strand to complete a beta-sandwich analogous to the UNC5b template structure. This region includes residue His-270, which was shown to be functionally important by mutagenesis. There is a chance that this residue comes close to the autocleavage site and therefore is involved in catalytic activity. Atoms are colored by their element: carbon, white; oxygen, red; and nitrogen, blue. This figure was prepared with PyMOL.
(TIF)

Figure S4 ***Role of His-252 in stabilizing the protein structure.*** Residue His-252 is located at the end of a beta-strand, which is part of a five stranded beta sheet. The side-chain hydrogen bonds with the carbonyl oxygen of Glu-285, located in the last strand of the sheet. This hydrogen bond orients the side-chain of Glu-285 such that it forms a salt bridge to Lys-509 in the CARD domain in exactly the same way as in the template structure UNC5b. A second hydrogen bond is formed with the backbone nitrogen of a neighboring residue, Ile-254. As histidine is encountered in different protonation states, hydrogen atoms have been added as green spheres in this figure. Hydrogen bonds are emphasized by dashed lines, which connect the heavy atom acceptor with the hydrogen atom. Distances are given in units of Ångström. This figure was prepared with PyMOL.
(TIF)

Figure S5 ***Model of CARD8 autoprocessing.*** Upon protein folding, the CARD and the ZU5-like domains of CARD8 interact with each other. This causes the conformational strain necessary for protein autocleavage. The two cleaved fragments remain associated after cleavage.
(TIF)

Methods S1 **Supporting Methods.**
(DOC)
