# Supplementary material for: CARD8 and NLRP1 Undergo Autoproteolytic Processing through a ZU5-Like Domain
Source: PLoS One. 2011 Nov 8;6(11):e27396. doi: 10.1371/journal.pone.0027396 (PMC3210808; doi:10.1371/journal.pone.0027396)
Supplement: Methods S1 — Supporting Methods. (DOC) [file pone.0027396.s006.doc]

**Supporting Information**

**Figure S1. *MS2 spectra of band 1 and 2.*** MS2 spectra for peptide VEPFYAVLESPSF from Gel Band 1 with MH+ 1484.7313, m/z 742.8693 and MS2 spectra for peptide SLMGILLR from Gel Band 2 with MH+ 918.5447, m/z 459.7760. b ions (blue) and y ions (red) are corresponding to tandem CID fragmentation of peptides matching the theoretical fragmentation pattern. Yellow M shows the oxidation of methionine.

**Figure S2. *Complete multiple sequence alignment of FIIND domains.*** High residue conservation is found across the whole domain. Sequences are sorted by percentage identity to the first listed protein, Human_card8. The most remote homolog is given by the death effector domain of dog, Dog_ded, which shares 47% identical residues with Human_card8. In a certain sense the opossum FIIND domain presents an outlier since it is equipped with an insertion of 41 residues at the C-terminus. As this fragment is absent in Human_card8, secondary structure prediction is not shown in the ‘SSPred’ row.

**Figure S3. *Complete model of the FIIND and CARD domains of human CARD8 protein.*** The model is based on the structure of UNC5b, deposited in PDB with accession code 3g5b. The FIIND domain consists of two domains, at the N-terminus a ZU5-like domain (green) followed by a UPA domain (blue). The CARD8 protein is terminated by a CARD domain (magenta). The center of the figure displays in ball-and-stick mode the SFS motif given by residues Ser-295, Phe-296, and Ser-297. Furthermore, residues Glu-240, Glu-242, His-252, His-270, Glu-279, His-280, and His-333 were selected for mutagenesis studies, they are colored orange in the MSA (Figure 2C and Supplementary Figure 2) and are also visualized as ball-and-stick representations. Autocleavage takes place at the C-terminus of the ZU5-like domain right before the last beta strand, which has been shown to be crucial in UNC5b. (In this figure, this beta strand has been rendered as an arrow for reasons of clarity even though the model lacks a beta sheet containing the strand.) A certain portion of the model lacks a confident structural prediction and has been rendered black. We assume this part forms another beta-strand to complete a beta-sandwich analogous to the UNC5b template structure. This region includes residue His-270, which was shown to be functionally important by mutagenesis. There is a chance that this residue comes close to the autocleavage site and therefore is involved in catalytic activity. Atoms are colored by their element: carbon, white; oxygen, red; and nitrogen, blue. This figure was prepared with PyMOL.

**Figure S4. *Role of His-252 in stabilizing the protein structure.*** Residue His-252 is located at the end of a beta-strand, which is part of a five stranded beta sheet. The side-chain hydrogen bonds with the carbonyl oxygen of Glu-285, located in the last strand of the sheet. This hydrogen bond orients the side-chain of Glu-285 such that it forms a salt bridge to Lys-509 in the CARD domain in exactly the same way as in the template structure UNC5b. A second hydrogen bond is formed with the backbone nitrogen of a neighboring residue, Ile-254. As histidine is encountered in different protonation states, hydrogen atoms have been added as green spheres in this figure. Hydrogen bonds are emphasized by dashed lines, which connect the heavy atom acceptor with the hydrogen atom. Distances are given in units of Ångström. This figure was prepared with PyMOL.

**Figure S5. *Model of CARD8 autoprocessing.*** Upon protein folding, the CARD and the ZU5-like domains of CARD8 interact with each other. This causes the conformational strain necessary for protein autocleavage. The two cleaved fragments remain associated after cleavage.

**Supporting Methods.**

**Protein Identification from Coomassie Stained Gel Bands.**

***In-gel protein digestion and sample preparation for LC/MS Analysis:*** SDS-PAGE Coomassie stained gel bands corresponding to bands 1 and 2 were cut to 1mm x 1mm pieces and transferred to separate new ethanol-rinsed 1.5 mL polypropylene centrifuge tubes (Eppendorf) and were de-stained. Following vacuum drying, proteins were reduced and alkylated at final concentration of 5 mM DTT and 15mM iodoacetamide prior to digestion with Trypsin at a final concentration of 25 ng/μl in 50 mM ammonium bicarbonate for 1 hour on ice and additionally 16 hours more at 37°C using a shaking incubator to ensure complete digestion. Digested tryptic peptides were extracted from gels by adding 80 μl of 5% Formic Acid in water, sonicated 10 min in water bath and then flowed by 4 time extraction by 50% aceto nitrile in 5% Formic Acid in water and once in 70% and 100% aceto nitrile. All extracted peptides were pooled together in an ethanol-rinsed Eppendorf tube and were vacuum dried and re-dissolved in 15 μl of 0.1% TFA. Tryptic peptides were then concentrated and desalted using a Millipore C18 Zip Tip (Millipore). The eluents were then vacuum dried and samples were re-dissolved in 25 μl of LC/MS loading buffer (2% acetonitrile in 0.1% formic acid in water).

***Protein Identification using 1D LC/MS/MS:*** 5 μl of tryptic digested samples were loaded into an automated Nano LC- LTQ MS/MS (Thermo Scientific, Waltham, MA), using an Eksigent Nano 2D LC system, a switch valve, a C18 trap column (Agilent, Santa Clara, CA), and a capillary reversed-phased column (15 cm in length, 100 mm id) packed with 5 mm, Magic C18 AQ resin (Michrom) with an ADVANCE ESI source (Michrom), which is used to ionize the peptides as they elute from the RP column, using a linear gradient elution from buffer A (2% acetonitrile in H_2_O plus 0.1% formic acid) to 15% buffer A plus 85% buffer B (ACN plus 0.1% formic acid) in 45 min. The LC/MS run was operated in the data- dependent mode. Data on the four strongest ions above an intensity of 50x10e4 were collected with dynamic exclusion enabled and the collision energy set at 35 %).

***Protein Identification and data analysis:*** The MS/MS spectra were analyzed by Sorcerer Enterprise v.4.0.4 release (Sage-N Research Inc.) with SEQUEST algorithm as the search program for peptide/protein identification. SEQUEST was set to search the target-decoy ipi.Human.v3.73 database containing protein sequences using trypsin as the enzyme with the allowance of up to 2 missed cleavages, Semi-tryptic search and precursor mass tolerance of 1.5 amu. Differential search includes 16 Da for methonine oxidation and 57 Da for cysteines to account for carboxyamidomethylation in case of alkylation of cysteines. The search results were viewed, sorted, filtered, and statically analyzed by using comprehensive proteomics data analysis software, Peptide/Protein prophet v.4.4.0 (ISB). To minimize false-positive identifications by MS/MS, we used the minimum trans-proteomic pipeline (TPP) probability score for proteins and peptides, each set at 0.95. We also set thresholds for cross correlation (Xcorr) scores for filtered peptides to1.5, 2.0, and 2.5 for 1+, 2+, and 3+ charged digested peptides, respectively. The Gene Ontology analysis of identified proteins was analyzed using Gene Ontology and also using QTools, an open source in-house developed tool for automated differential peptide/protein spectral count analysis.
